# Roles of Mitochondrial Serine Hydroxymethyltransferase 2 (SHMT2) in Human Carcinogenesis

**DOI:** 10.7150/jca.60170

**Published:** 2021-08-08

**Authors:** Yuanyuan Zeng, Jie Zhang, Mengmeng Xu, Fuxian Chen, Ruidong Zi, Jicheng Yue, Yanan Zhang, Nannan Chen, Y. Eugene Chin

**Affiliations:** 1 Institutes of Biology and Medical Sciences, Soochow University, Suzhou 215123, Jiangsu, China.; 2 Department of Respiratory Medicine, the First Affiliated Hospital of Soochow University, Suzhou 215006, Jiangsu, China.

**Keywords:** Serine hydroxymethyltransferase 2 (SHMT2), human carcinogenesis, predictive biomarker, cell proliferation, tumour growth

## Abstract

In the last few years, cellular metabolic reprogramming has been acknowledged as a hallmark of human cancer and evaluated for its crucial role in supporting the proliferation and survival of human cancer cells. In a variety of human tumours, including hepatocellular carcinoma (HCC), breast cancer and non-small-cell lung cancer (NSCLC), a large amount of carbon is reused in serine/glycine biosynthesis, accompanied by higher expression of the key glycine synthetic enzyme mitochondrial serine hydroxymethyltransferase 2 (SHMT2). This enzyme can convert serine into glycine and a tetrahydrofolate-bound one-carbon unit, ultimately supporting thymidine synthesis and purine synthesis and promoting tumour growth. In tumour samples, elevated expression of SHMT2 was found to be associated with poor prognosis. In this review, the pivotal roles of SHMT2 in human carcinogenesis are described, highlighting the underlying regulatory mechanisms through promotion of tumour progression. In conclusion, SHMT2 may serve as a prognostic marker and a target for anticancer therapies.

## Introduction

**Figure 1:**
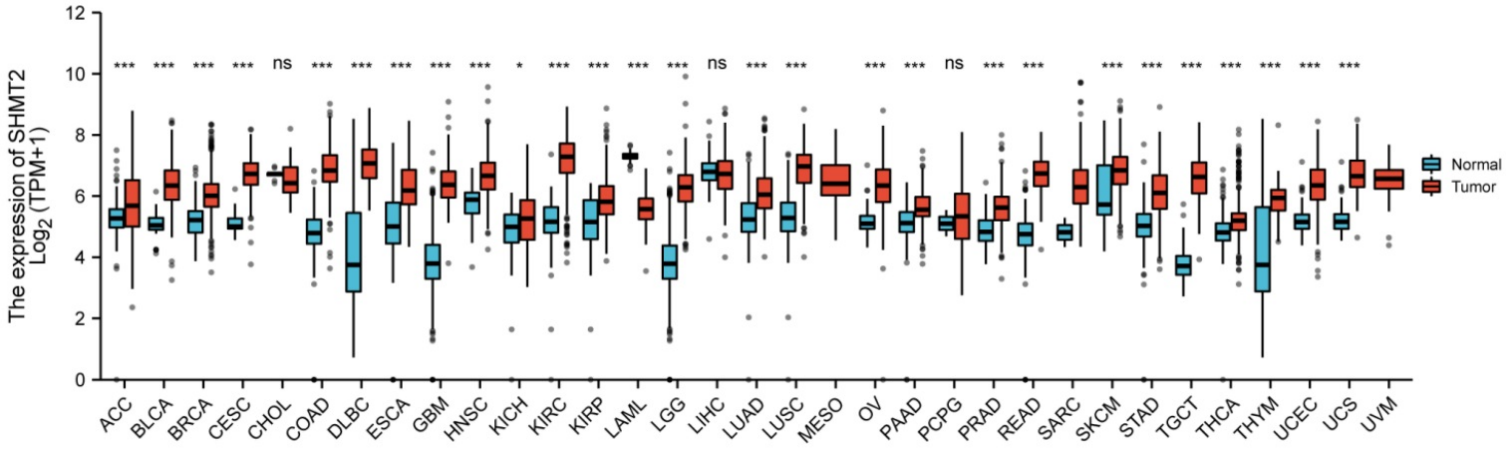
Different expression levels of SHMT2 in human tumour types from TCGA database (*p < 0.05, **p < 0.01, ***p < 0.001).

Cumulative evidence suggests that the serine and glycine biosynthetic pathways are linked to the biosynthesis of cellular components to sustain cell proliferation. Dysfunction of genes involved in the one-carbon unit biosynthetic pathway has been linked to increased risks of various human cancers 1, 2. SHMT2 is a key enzyme in one-carbon unit metabolism and catalyzes the conversion of serine and tetrahydrofolate (THF) to glycine and 5,10-methylenetetrahydrofolate (5,10-CH_2_-THF) 3. Specifically, SHMT2 expression exhibits a significant correlation with a wide variety of human phenotypes, including neural tube defects and childhood acute leukaemia. To assess the relationship between SHMT2 and human cancers, we analysed *SHMT2* expression with public RNA-seq data from TCGA in multiple malignancies. As shown in Figure 1, *SHMT2* was significantly elevated in more than 85% of cancer types compared with normal tissues. Similarly, a series of studies have reported that SHMT2 expression is increased in a subset of human cancers including colon cancer, breast cancer, lung cancer, ovarian cancer and prostate cancer. Recently, an advanced understanding of the mechanisms of SHMT2 in human carcinogenesis has provided novel opportunities for drug development, dietary intervention and therapeutic targets 4.

## Identification and Characterization of SHMT2

**Figure 2:**
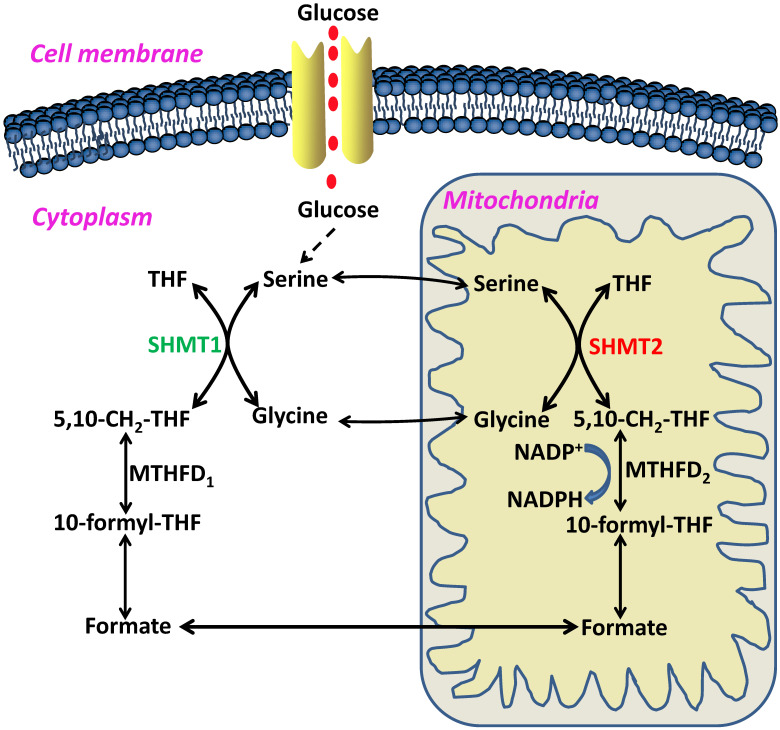
Schematic overview of serine/glycine metabolism in the cytoplasm and mitochondria. THF, tetrahydrofolate; 5,10-CH2-THF, 5,10-methylenetetrahydrofolate; MTHFD2, methylenetetrahydrofolate dehydrogenase 2; NADPH, nicotinamide adenine dinucleotide phosphate; 10-formyl-THF, 10-formyl-tetrahydrofolate; MTHFD1, methylenetetrahydrofolate dehydrogenase 1; SHMT1, serine hydroxymethyltransferase 1; SHMT2, serine hydroxymethyltransferase 2.

**Figure 3:**
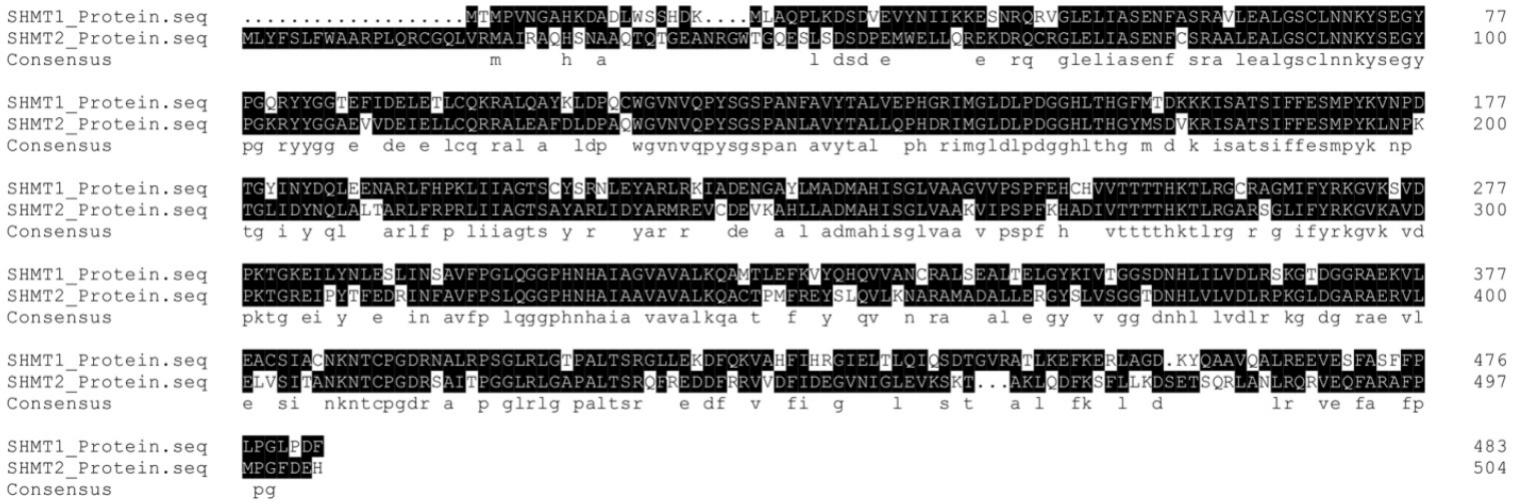
Sequence of the protein SHMT2 aligned with SHMT1. Identical residues are highlighted in dark.

In 1981, Stauffer and his colleagues first cloned the *glyA* gene, which encodes the *E. coli* SHMT protein, a pyridoxal 5'-phosphate (PLP) dependent enzyme 5. The reverse reaction between glycine and serine catalyzed by SHMT was named the 'futile cycle', and critical roles of the reaction product 5-formylTHF have been found in maintaining one-carbon unit homeostasis 6, 7 (Figure 2). In humans, SHMT consists of two isoforms, SHMT1, which is located in the cytosol (cSHMT), and SHMT2, which is located in the mitochondria (mSHMT). These two isoforms display an amino acid sequence identity of approximately 66% (Figure 3). In addition, SHMT1 forms a tetramer in the cytoplasm, while SHMT2 exists in a dimer-to-tetramer transition depending on pyridoxal 5'-phosphate (PLP) binding 8, 9. In fact, SHMT2 can encode SHMT2α, a second transcript that lacks the import signal location to the mitochondria and is localized in the cytoplasm. Due to its important role in the cycle of one-carbon unit, SHMT2 is ubiquitous and highly conserved in all organisms.

## Deregulation of SHMT2 in Various Human Cancers

Many studies have demonstrated the crucial roles of SHMT2 in maintaining normal methylation patterns, DNA stability and genetic variation 10, 11. SHMT2 maps to 12q13 with all intron/exon splice junctions conforming to the GT/AG rule 6, 12. Several studies have suggested that the encoded product is expressed predominately in the mitochondria and primarily responsible for glycine synthesis in the cell 13. While SHMT1 maps to 17p11.2 and is expressed as multiple splice variants with 330 nucleotides of the 5'-untranslated region. There are several consensus DNA recognition sites for transcription factor binding in the 5'-promoter region of the mSHMT gene. Sequence analysis of the *mSHMT* gene revealed two Myc consensus binding sites in its promoter. Transcriptional activation of *SHMT2* is regulated by the oncogenic transcription factor c-Myc 11. Moreover, transcriptional regulatory proteins, including Sp-1, AP-2, and PEA3, may control endogenous expression of SHMT2 for further investigation. Primer extension analysis and 5'-RACE analysis reveal that transcription initiation of cSHMT occurs at multiple sites. Northern blot analysis of multiple tissues suggests that cSHMT displays cell-specific splicing patterns. Multiple forms of cSHMT are present in MCF-7 cells, whereas a single form is expressed in SH_SY5Y cells 7. Although SHMT1 and SHMT2 catalyze the same biochemical reactions, they play different biological roles in tumors. Recent studies have more focused on single nucleotide polymorphism (SNP) of SHMT1 and indicated that the SNPs may associate with cancers 14, 15. However, meta-analysis by Wang Q et al about roles of *SHMT1* gene in various cancer types indicated the inconsistent roles of SHMT1 in multiple tumor procession 16. Then in view of the consistent roles of SHMT2 and complex regulatory mechanisms in human tumour, we reviewed the current studies in multiple cancers for a comprehensive understanding of SHMT2.

### Hepatocellular carcinoma (HCC)

Intracellular biosynthesis of glycine catalysed by SHMT2 is essential for hepatocyte growth and proliferation. Knockdown of SHMT2 expression worsened hepatic ischaemia-reperfusion and delayed liver regeneration after partial hepatectomy 17, 18. However, aberrant SHMT2 promoted changes in metabolism that enhanced the survival capacities of tumour cells to survive in the ischaemic microenvironment. Lee et al. found that SHMT2 was significantly upregulated at both the mRNA and protein levels in three human HCC cell lines (Hep3B, HepG2 and Huh-7) compared with an immortalized normal liver cell line (THLE2) 19. Inhibition of SHMT2 led to reduced cell proliferation and tumorigenicity in liver cancer cells. In addition, Huh-7 cells with downregulated SHMT2 expression failed to form tumours in a human tumour xenograft mouse model, whereas SHMT2 overexpression enhanced liver cancer cell survival and proliferation 19, 20. Moreover, an increasing number of studies have demonstrated that SHMT2 is overexpressed in the tissues of hepatocellular and intrahepatic biliary carcinoma patients and that a higher SHMT2 level is clinically related to a worse survival outcome than a lower level of SHMT2, suggesting that SHMT2 could be used as a potential prognostic biomarker in HCC 20, 21.

### Gastrointestinal cancer

The role of the mitochondrial metabolic enzyme SHMT2 in gastrointestinal cancer has been reported in recent years for its function in providing one-carbon unit of serine-glycine conversion involved in one-carbon unit metabolism. SHMT2 acetylation was decreased in human colonic epithelial cell lines, which eventually promoted colorectal carcinogenesis 22. Later studies confirmed upregulated SHMT2 expression in colon cancer tissues 23 and showed that decreased invasion and inhibition of proliferation of colorectal carcinoma cells were accompanied by downregulation of SHMT2 expression* in vitro* 24. Clinically, SHMT2 was overexpressed in gastric cancer, oesophageal cancer and colorectal cancer (CRC) tissues at both the mRNA and protein levels, as measured by qRT-PCR and immunohistochemistry assays, compared to paired normal adjacent tissues. Furthermore, survival and correlation analysis revealed that SHMT2 expression was an independent prognostic factor for recurrence-free survival and disease-specific survival in gastric cancer, oesophageal cancer and CRC 25. Similarly, Shi et al. assessed SHMT2 expression in 130 gastric cancer tissues using immunohistochemistry and demonstrated that SHMT2 expression was enhanced compared to that in adjacent tissues. Patients with higher expression of SHMT2 showed significantly worse outcomes, which supported that SHMT2 acted as an attractive target for gastrointestinal cancer chemotherapy 26.

### Glioma

SHMT2 expression was also associated with the survival of brain cancer cells within the ischaemic zones of gliomas 27. Overexpression of SHMT2, on the one hand, restricted the activity of pyruvate kinase and reinforced glycolytic progression, thus lessening oxygen demand and assisting cancer cells in enduring and surviving in an ischaemic tumour microenvironment. On the other hand, the enhanced glycine production also rendered these cells sensitive to inhibition by the glycine cleavage system. Intriguingly, SHMT2 expression can also be induced in glioblastoma cell lines (LN-308, LNT-229 and G55 cells); this molecule cooperates with phosphoglycerate dehydrogenase (PHGDH) to support glioblastoma cell adaption in hypoxic conditions 28, 29. In line with that, clinical detection of SHMT2 expression by immunohistochemistry revealed that it was highly upregulated in glioma tissues compared to that in the control group. In addition, a Kaplan-Meier analysis showed that higher SHMT2 expression was implicated in poorer progression-free survival (PFS) and overall survival (OS), although the data failed to link the expression of SHMT2 with drug resistance mechanisms in glioma patients 30, 31.

### Breast cancer

Emerging studies have demonstrated the pivotal roles of SHMT2 protein in breast cancer progression 32. As shown in the work of Li et al., subclones of MDA-MB-231 cell lines with elevated one-carbon unit biosynthesis activity displayed increased metastatic potential 33. Knockdown of SHMT2 expression potently repressed the proliferation of metastatic subclones *in vitro*. Moreover, impaired cancer cell proliferation due to the inhibition of SHMT2-induced metabolic reprogramming was further validated in animal models. Compared to that in the matched noncancerous tissues, SHMT2 protein expression was also upregulated in clinical breast cancer tissues. Consistently, there was a significant correlation between the expression level of SHMT2 and breast cancer grade. More importantly, both primary and metastatic tumour growth was suppressed in the SHMT2 knockdown group. Similarly, another study showed that SHMT2 protein expression was detected in 128 breast cancer cases and associated with tumour aggressiveness (TNM staging and Elson grade) in a dose-dependent manner. Stratified analysis results indicated that SHMT2 was helpful in predicting outcomes, especially in oestrogen receptor (ER)-negative breast cancer patients. In cases involving stage IIb breast cancer, chemotherapy significantly extended survival time among patients with higher SHMT2 expression 34. Although this molecule is considered a prognostic biomarker of breast cancer, other studies have reported that abnormal expression of SHMT2 may predict negative prognosis 35, 36. Nevertheless, there was no association with age, sex, lymph node status, TNM stage or vascular invasion status 37, 38.

Notably, recent studies have linked SHMT2 overexpression with resistance to clinical therapy in breast cancer. Once exposed to lapatinib, cells with elevated SHMT2 could attenuate the toxicity of reactive oxygen species (ROS) and survive 39. In addition, though upregulating the expression of the antioxidant enzymes HMOX1, SHMT2 and SLC7A11, breast cancer cells exhibited resistance to paclitaxel through the EIF2AK3/EIF2AK4-pEIF2S1-ATF4 axis 40.

Collectively, these results indicated that SHMT2 may be a valuable prognostic biomarker in breast cancer. Furthermore, SHMT2 may serve as a potential therapeutic target for breast cancer treatment and drug discovery 37, 41.

### Other cancers

The SHMT2 expression level was not only increased and implicated in poor prognosis in lung cancer 42, 43 but was also associated with idiopathic pulmonary fibrosis (IPF) 44. Based on application of western blotting and immunohistochemistry staining, the expression of SHMT2 was found to be increased in response to TGF-β *in vitro* and in lung tissues obtained from four patients with IPF compared with expression in healthy donors. Furthermore, upregulated SHMT2 expression increased the aggressiveness of prostate cancer cells 45. Additionally, a study of osteoarthritis showed that SHMT2 was overexpressed in osteoarthritis patients and involved in the pathogenesis of osteoarthritis by modulating one-carbon unit metabolism 46.

Altogether, SHMT2 acts as an important enzyme in one-carbon unit metabolism pathway. Different studies suggest the diverse functions of SHMT2 in the majority of tumours. A genome-wide oncogenic screening strategy and RNAi screen pioneered by Lee's group identified SHMT2 as a candidate oncogenic driver gene across multiple solid tumour types 47. Subsequent functional assays showed that SHMT2 was necessary for tumour cell survival across a panel of 32 cancer cell lines. A series of experimental and functional analyses revealed the oncogenic properties of SHMT2 in promoting cancer cell survival and tumour growth* in vivo*.

## Mechanisms Underlying the Oncogenic Role of SHMT2 in Human Carcinogenesis

SHMT2 has a role in folate metabolism, as it provides active one-carbon unit contributing to the biosynthesis of nucleotides and proteins involved in tumour growth. Although the physiological function of the isozyme has been widely studied, the specific mechanisms of the oncogenic role of SHMT2 in human carcinogenesis remain to be further determined.

SHMT2 is primarily involved in the production of glycine and 5,10-CH_2_-THF 48. In mitochondria, SHMT2-dependent production of methylene-THF contributes to mitochondrial NADPH generation and redox balance during hypoxia 29. Hypoxia plays critical roles in metastasis and angiogenesis by upregulating the expression of glycolysis-related enzymes, including HK2, LDHA and PDK1. The upregulated expression of these enzymes suppresses entry of pyruvate into the TCA cycle, thus reducing ROS generation and promoting tumour growth. Previous studies have proposed that SHMT2 is a transcriptional target of Myc, illustrating an intrinsic link between tumorigenesis and cellular metabolism 11, 49. Myc amplification was shown to promote upregulation of SHMT2 expression in a hypoxia-inducible factor-1α (HIF-1α)-dependent manner, resulting in increased production of NADPH from NADP^+^, restrained cellular reactive oxygen species, and enhanced tumour cell survival 29. SHMT2 knockdown significantly inhibited xenograft tumour growth and aggravated ischaemia-reperfusion injury in mice through the ROS/JNK/P53 signaling pathway 17, 29. Moreover, SHMT2 can activate the Akt/mTOR signaling pathway by its metabolic product, which facilitates hepatocyte cell regeneration 18. SHMT2 expression can also be upregulated through the IL-6/JAK2/STAT3 regulatory pathway in human prostate carcinoma LNCaP cells, speeding up the cell transition towards more advanced stages 45.

Studies on noncoding RNAs in tumours have been increasing in recent years, and it was also reported that SHMT2 could be directly and negatively regulated by miR-370 in human articular chondrocytes and miR-615-5p in HCC cells 46. In NSCLC cells, long noncoding RNA (lncRNA) Gm15290 exerted a pro-oncogene effect by negatively regulating the expression of miR-615-5p and thus increasing the protein levels of miR-615-5p target genes, including SHMT2 50. In addition, the long noncoding RNA LINC01234 promoted colon cancer cell proliferation through the LINC01234-miR642a-5p-SHMT2 axis 24. Furthermore, it was found that circular RNA 0072995 directly targets miR-149-5p, thereby upregulating the expression of its downstream gene SHMT2 and enhancing breast tumour cell proliferation *in vitro* and *in vivo* 51.

**Figure 4:**
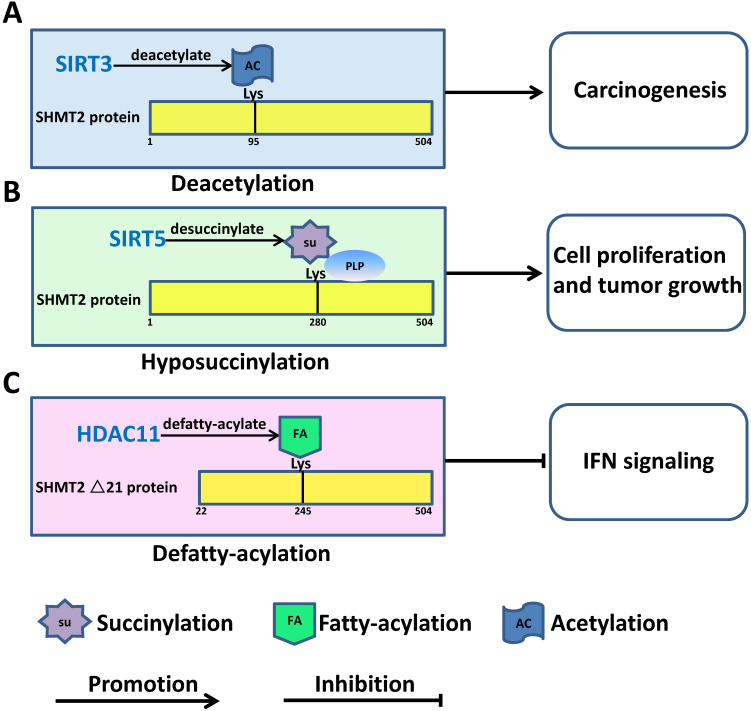
Post-translational modification of SHMT2 regulates human cancer progression. **A.** Deacetylation of SHMT2 by SIRT3 at K95 promotes colorectal carcinogenesis. **B.** Desuccinylation of SHMT2 by SIRT5 at K280 promotes cancer cell proliferation and tumour growth. **C.** Defatty acylation of SHMT2 by HDAC11 at K95 regulates type I interferon signaling.

In addition to interaction with signaling pathways or modulation by miRNA, inadequate SHMT denaturalization can lead to human cancer progression via post-translational modification. SHMT2 could be acetylated at lysine K95 in different cancer cells, including CRC cells. K95 acetylation inhibits SHMT2 enzymatic activity and promotes its degradation via macroautophagy. SIRT3, a deacetylase localized in the mitochondria, deacetylated SHMT2 and eventually increased its activity to promote cell proliferation and colorectal carcinogenesis 22 (Figure 4A). SHMT2 interacts with nicotinamide adenine dinucleotide (NAD^+^)-dependent lysine desuccinylase SIRT5 directly. Under metabolic stress, desuccinylation at lysine 280 of SHMT2 by SIRT5 enhances SHMT2 activity to accelerate cell metabolism and facilitate cell proliferation and tumour growth *in vivo* and* in vitro* 52-54 (Figure 4B). HDAC11 has been proven to be an efficient lysine defatty-acylase that directly removes fatty acyl groups from SHMT2. In MCF-7 and A549 cells, knockdown of HDAC11 significantly increased the fatty acylation level of SHMT2. Although SHMT2 fatty acylation does not affect its enzymatic activity, K245 is the major fatty acylation site of HDAC11. Upon treatment with IFNα, fatty-acylated SHMT2 was recruited to late endosomes/lysosomes to deubiquitinate IFNαR1 and activate IFN signaling 55-57 (Figure 4C).

## Perspectives

In cellular metabolism, glycine metabolism is a key step in the proliferation and survival of human cancer cells 58. SHMT2 is an oncogene, and thus, it may serve as a promising target for anticancer therapy. The identification of selective SHMT2 inhibitors could be an innovative and promising approach for the treatment of various cancers. Although SHMT2 is a difficult target for inhibition, as it is located in mitochondria, further drug discovery efforts are warranted. There are already preclinical studies exploring and evaluating the potential efficacy of inhibitors targeting SHMT2 and cytosolic C1 enzymes 59-61, among which AGF347 showed a satisfying and compelling anti-tumour effects *in vivo* in both early-stage and advanced stage MIA PaCa-2 pancreatic tumour xenografts^62^. Notably, anticancer models using SHMT2 knockout for treating human cancer may overestimate the anticancer effect of SHMT2 63, 64. The deletion of SHMT2 will inhibit translation in the mitochondria, whereas the effects of the drug may not achieve the depletion of one-carbon unit required to produce the same phenotype 65, 66. In summary, tumour cells become more aggressive under internal and external stresses. The prevention of tumour development, metastasis and recurrence by the perturbation of specific SHMT2 inhibitors requires further exploration.

## Abbreviations

NSCLC: non-small-cell lung cancer
HCC: hepatocellular carcinoma
SHMT2: serine hydroxymethyltransferase 2
THF: tetrahydrofolate
5,10-CH_2_-THF: 5,10-methylenetetrahydrofolate
PLP: pyridoxal 5'-phosphate
CRC: colorectal cancer
IPF: idiopathic pulmonary fibrosis
HIF-1α: hypoxia-inducible factor-1α
